# Uterine smooth muscle tumours with uncertain malignant potential: reproductive and clinical outcomes in patients undergoing fertility-sparing management

**DOI:** 10.1093/hropen/hoaf009

**Published:** 2025-03-03

**Authors:** Umberto Leone Roberti Maggiore, Francesco Fanfani, Giovanni Scambia, Ilaria Capasso, Emanuele Perrone, Giuseppe Parisi, Gian Franco Zannoni, Francesca Falcone, Alessandra Di Giovanni, Mario Malzoni, Anna Myriam Perrone, Francesco Mezzapesa, Pierandrea De Iaco, Simone Garzon, Pier Carlo Zorzato, Stefano Uccella, Fabio Barra, Stefano Bogliolo, Simone Ferrero, Veronica Iannuzzi, Dorella Franchi, Tommaso Bianchi, Tommaso Grassi, Robert Fruscio, Giulia Vittori Antisari, Giovanni Roviglione, Marcello Ceccaroni, Fulvio Borella, Stefano Cosma, Alberto Revelli, Jvan Casarin, Anna Giudici, Fabio Ghezzi, Matteo Marchetti, Giulia Spagnol, Roberto Tozzi, Francesca Filippi, Michela Molgora, Giovanna Scarfone, Biagio Paolini, Stefano Fucina, Valentina Chiappa, Antonino Ditto, Giorgio Bogani, Francesco Raspagliesi

**Affiliations:** Gynecologic Oncology Unit, Fondazione IRCCS Istituto Nazionale dei Tumori, Milan, Italy; Department of Women, Children, and Public Health Sciences, Fondazione Policlinico Universitario A. Gemelli IRCCS, Rome, Italy; Department of Women, Children, and Public Health Sciences, Fondazione Policlinico Universitario A. Gemelli IRCCS, Rome, Italy; Department of Women, Children, and Public Health Sciences, Fondazione Policlinico Universitario A. Gemelli IRCCS, Rome, Italy; Department of Women, Children, and Public Health Sciences, Fondazione Policlinico Universitario A. Gemelli IRCCS, Rome, Italy; Department of Women, Children, and Public Health Sciences, Fondazione Policlinico Universitario A. Gemelli IRCCS, Rome, Italy; Department of Women, Children, and Public Health Sciences, Fondazione Policlinico Universitario A. Gemelli IRCCS, Rome, Italy; Center for Advanced Endoscopic Gynecological Surgery, Endoscopica Malzoni, Avellino, Italy; Center for Advanced Endoscopic Gynecological Surgery, Endoscopica Malzoni, Avellino, Italy; Center for Advanced Endoscopic Gynecological Surgery, Endoscopica Malzoni, Avellino, Italy; Department of Medical and Surgical Sciences, University of Bologna, Bologna, Italy; Division of Oncologic Gynecology, IRCCS Azienda Ospedaliero-Universitaria Di Bologna, Bologna, Italy; Department of Medical and Surgical Sciences, University of Bologna, Bologna, Italy; Division of Oncologic Gynecology, IRCCS Azienda Ospedaliero-Universitaria Di Bologna, Bologna, Italy; Department of Medical and Surgical Sciences, University of Bologna, Bologna, Italy; Division of Oncologic Gynecology, IRCCS Azienda Ospedaliero-Universitaria Di Bologna, Bologna, Italy; Unit of Obstetrics and Gynecology, Department of Surgery, Dentistry, Pediatrics, and Gynecology, AOUI Verona, University of Verona, Verona, Italy; Unit of Obstetrics and Gynecology, Department of Surgery, Dentistry, Pediatrics, and Gynecology, AOUI Verona, University of Verona, Verona, Italy; Unit of Obstetrics and Gynecology, Department of Surgery, Dentistry, Pediatrics, and Gynecology, AOUI Verona, University of Verona, Verona, Italy; Unit of Obstetrics and Gynecology, P.O. “Ospedale del Tigullio”-ASL4, Chiavari (Genoa), Italy; Unit of Obstetrics and Gynecology, P.O. “Ospedale del Tigullio”-ASL4, Chiavari (Genoa), Italy; Department of Neurosciences, Rehabilitation, Ophthalmology, Genetics, Maternal and Child Health (DiNOGMI), University of Genoa, Genoa, Italy; Academic Unit of Obstetrics and Gynecology, IRCCS Ospedale Policlinico San Martino, Genoa, Italy; Preventive Gynecology Unit, Division of Gynecology, European Institute of Oncology IRCCS, Milan, Italy; Preventive Gynecology Unit, Division of Gynecology, European Institute of Oncology IRCCS, Milan, Italy; UO Gynecology, IRCCS San Gerardo dei Tintori, Monza, Italy; Department of Medicine and Surgery, University of Milan-Bicocca, Milan, Italy; UO Gynecology, IRCCS San Gerardo dei Tintori, Monza, Italy; Department of Medicine and Surgery, University of Milan-Bicocca, Milan, Italy; UO Gynecology, IRCCS San Gerardo dei Tintori, Monza, Italy; Department of Medicine and Surgery, University of Milan-Bicocca, Milan, Italy; Unit of Obstetrics and Gynecology, IRCCS Sacred Heart Hospital Don Calabria, Negrar (Verona), Italy; Unit of Obstetrics and Gynecology, IRCCS Sacred Heart Hospital Don Calabria, Negrar (Verona), Italy; Unit of Obstetrics and Gynecology, IRCCS Sacred Heart Hospital Don Calabria, Negrar (Verona), Italy; Gynecology and Obstetrics Unit 1, Department of Surgical Sciences, City of Health and Science University Hospital, University of Turin, Turin, Italy; Gynecology and Obstetrics Unit 1, Department of Surgical Sciences, City of Health and Science University Hospital, University of Turin, Turin, Italy; Gynecology and Obstetrics Unit 2, Department of Surgical Sciences, City of Health and Science University Hospital, University of Turin, Turin, Italy; Obstetrics and Gynecology Unit, Women’s and Children Hospital, University of Insubria, Varese, Italy; Obstetrics and Gynecology Unit, Women’s and Children Hospital, University of Insubria, Varese, Italy; Obstetrics and Gynecology Unit, Women’s and Children Hospital, University of Insubria, Varese, Italy; Unit of Gynecology and Obstetrics, Department of Women and Children’s Health, University of Padua, Padua, Italy; Unit of Gynecology and Obstetrics, Department of Women and Children’s Health, University of Padua, Padua, Italy; Unit of Gynecology and Obstetrics, Department of Women and Children’s Health, University of Padua, Padua, Italy; Department of Obstetrics, Gynecology and Neonatology, Fondazione IRCCS Ca' Granda Ospedale Maggiore Policlinico, Milan, Italy; Department of Obstetrics, Gynecology and Neonatology, Fondazione IRCCS Ca' Granda Ospedale Maggiore Policlinico, Milan, Italy; Department of Clinical Sciences and Community Health, Università degli Studi di Milano, Milan, Italy; Department of Obstetrics, Gynecology and Neonatology, Fondazione IRCCS Ca' Granda Ospedale Maggiore Policlinico, Milan, Italy; 1st Pathology Division, Department of Pathology and Laboratory Medicine, Fondazione IRCCS Istituto Nazionale dei Tumori, Milan, Italy; Gynecologic Oncology Unit, Fondazione IRCCS Istituto Nazionale dei Tumori, Milan, Italy; Gynecology and Obstetrics Unit 1, Department of Surgical Sciences, City of Health and Science University Hospital, University of Turin, Turin, Italy; Gynecologic Oncology Unit, Fondazione IRCCS Istituto Nazionale dei Tumori, Milan, Italy; Gynecologic Oncology Unit, Fondazione IRCCS Istituto Nazionale dei Tumori, Milan, Italy; Gynecologic Oncology Unit, Centro di Riferimento Oncologico, National Cancer Institute, Aviano, Italy; Gynecologic Oncology Unit, Fondazione IRCCS Istituto Nazionale dei Tumori, Milan, Italy; Gynecologic Oncology Unit, Fondazione IRCCS Istituto Nazionale dei Tumori, Milan, Italy

**Keywords:** fertility, leiomyoma, reproductive outcomes, sarcoma, survival, STUMP, uterine tumour

## Abstract

**STUDY QUESTION:**

Can patients with uterine smooth muscle tumours of uncertain malignant potential (STUMP) be effectively and safely managed with fertility-sparing treatment?

**SUMMARY ANSWER:**

This multicentre retrospective study demonstrates that fertility-sparing management for patients diagnosed with STUMP is both feasible and safe.

**WHAT IS KNOWN ALREADY:**

Few studies, involving a limited number of patients, have investigated fertility-sparing management for STUMP in women with future pregnancy aspirations.

**STUDY DESIGN, SIZE, DURATION:**

This multicentre retrospective study was conducted in collaboration with 13 Italian institutions specializing in gynaecologic oncology. The primary objective was to evaluate the reproductive outcomes of the included patients, while the secondary objective was to analyse their clinical outcomes.

**PARTICIPANTS/MATERIALS, SETTING, METHODS:**

A total of 106 patients with a histological diagnosis of STUMP who underwent fertility-sparing treatment for uterine tumours were included. Patient data were collected from 13 referral centres across Italy, and reproductive and clinical outcomes were documented during follow-up. The median (range) length of follow-up was 48 (7–191) months.

**MAIN RESULTS AND THE ROLE OF CHANCE:**

Of the 106 patients, 47 (44.3%) patients actively tried to conceive after fertility-sparing surgery, and 27 of them (57.4%) achieved a pregnancy. Among the patients trying to conceive, 12 (25.5%) women had more than one pregnancy after surgery for STUMP. At follow-up, 23 (21.7%) out of the 106 women had a recurrence of uterine disease. Furthermore, a higher rate of recurrence was observed among patients who became pregnant (17 out of 27 women (63.0%)) compared with those who did not (6 out of 79 women (7.6%); *P* < 0.001). Only two cases (1.9%) of malignant relapse were recorded, and one patient with a leiomyosarcoma recurrence died.

**LIMITATIONS, REASONS FOR CAUTION:**

The primary limitation of this study is the inherent biases associated with its retrospective design.

**WIDER IMPLICATIONS OF THE FINDINGS:**

This multicentre retrospective study represents the largest case series to date examining the reproductive and clinical outcomes of patients undergoing conservative treatment for STUMP. The findings suggest that patients can be counselled on the feasibility and safety of fertility-sparing management, which should be considered by clinicians as both safe and effective.

**STUDY FUNDING/COMPETING INTEREST(S):**

This work was supported by Ricerca Corrente funds, Italian Ministry of Health. There are no competing interests.

**TRIAL REGISTRATION NUMBER:**

N/A.

WHAT DOES THIS MEAN FOR PATIENTS? This study examines whether women with a rare type of uterine tumour, called smooth muscle tumours of uncertain malignant potential (STUMP), can safely undergo treatment that preserves their fertility. STUMP tumours are difficult to classify as either clearly benign or malignant, making treatment decisions challenging, particularly for women who wish to have children.

The study was conducted by a group of gynaecologic oncology specialists from 13 Italian medical centres, including records of 106 women who were diagnosed with STUMP and who opted for fertility-sparing surgery, which removes the tumour while keeping the uterus intact.

The main findings of this study are as follows. (i) Pregnancy outcomes: among 47 women who actively tried to conceive after surgery, 27 (57.4%) became pregnant, and 12 had more than one pregnancy. (ii) Recurrence risk: about 22% of women had their tumours return, but in most cases, the recurrence was not cancerous. (iii) Cancer risk: only two women (1.9%) developed cancerous tumours, and one of them passed away from the disease.

This study suggests that fertility-preserving surgery is a reasonable and safe option for women with STUMP who wish to have children. While there is a risk of tumour recurrence, most cases remain non-cancerous. However, doctors should closely monitor patients over time to detect any signs of progression to cancer.

The findings provide valuable guidance for women diagnosed with STUMP, and their doctors, when discussing treatment options. The study emphasizes that fertility-sparing surgery should be performed in specialized centres with expertise in gynaecologic oncology to ensure the best outcomes.

## Introduction

**Figure 1.**
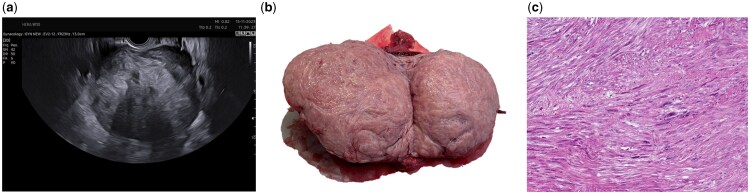
**STUMP at ultrasound and macroscopic and microscopic evaluations.** (**a**) A case of isoechoic-hyperchoic uterine STUMP, with well-defined outlines and shadowing; (**b**) macroscopic image of a STUMP lesion at gross examination; and (**c**) pathological detail of a STUMP: severe plurifocal (‘significant’) nuclear atypia in the absence of tumour necrosis, with irrelevant mitotic index. STUMP, uterine smooth muscle tumours with uncertain malignant potential.

Smooth muscle tumours of the uterus are the most common neoplasms of the female genital tract (Devereaux and Schoolmeester, 2019). By the age of 50, the cumulative incidence is estimated to range between 70% among white women and 80% among Black women (Baird *et al.*, 2003). These tumours can be classified as benign (leiomyomas), which constitute the vast majority, or malignant (leiomyosarcomas), based on three histopathological criteria: cytologic atypia, mitotic count, and tumour cell necrosis (Bell *et al.*, 1994). In addition, the 2014 World Health Organization (WHO) classification described smooth muscle tumours of unknown malignant potential (STUMPs) (Fig. 1) as tumours with pathological features that preclude a definitive diagnosis of leiomyosarcoma (i.e. diffuse cytologic atypia, tumour cell necrosis, and ≥10 mitoses per 10 high power fields), but that do not fulfil the criteria for leiomyoma (bland appearance, no tumour cell necrosis, and ≤4 mitoses per 10 high power fields) or its variants, raising concern about a potential malignant behaviour (Oliva *et al.*, 2014; Ip *et al.*, 2020). Patients with STUMPs typically present at a mean or median age of 41 to 48 years, with cases reported across a broad age range of 20 to 75 years (Gadducci and Zannoni, 2019). STUMPs account for approximately 2–5% of all uterine smooth muscle tumours and pose significant challenges in management due to their uncertain malignant potential (Bucuri *et al.*, 2024). Accurate preoperative diagnosis of uterine smooth muscle tumours is essential to determine the most appropriate treatment strategy, whether fertility-sparing or radical surgery, without compromising the prognosis in the event of malignancy. However, non-specific symptoms and inconclusive imaging, which often reveal overlapping features between benign and malignant lesions, make it difficult to differentiate among various types of myometrial tumours (Chiappa *et al.*, 2021). Although many STUMPs are benign and associated with favourable long-term outcomes, some may exhibit aggressive malignant behaviour, metastasize, and contribute to increased tumour-related mortality. A recent systematic review reported a recurrence rate of 21.5%, with ∼30% of recurrences involving malignant histological types, such as leiomyosarcoma (Bucuri *et al.*, 2024).

In recent years, there has been a significant trend towards delayed childbearing, with women in Western countries increasingly achieving their first pregnancy at older maternal ages (Mathews and Hamilton, 2014). Notably, smooth muscle tumours of the uterus are commonly diagnosed in women of reproductive age, particularly in their 30s and 40s (Zimmermann *et al.*, 2012). Consequently, there is growing demand for fertility-sparing procedures, necessitating a discussion with patients about the possibility of uterine preservation. The literature on conservative management of STUMPs in women desiring future pregnancies is limited, with only a small number of studies involving relatively few patients (Campbell *et al.*, 2016; Ha *et al.*, 2018; Karataşlı *et al.*, 2019; Şahin *et al.*, 2019; Huo *et al.*, 2020; Shim *et al.*, 2020; Ning *et al.*, 2021; Zhang *et al.*, 2021; Richtarova *et al.*, 2023; Borella *et al.*, 2024; Garg *et al.*, 2024).

Therefore, we designed this Italian multicentre retrospective study to investigate the reproductive and clinical outcomes of women who were histologically diagnosed with STUMP and who wished to receive fertility-sparing management.

## Materials and methods

A multicentre retrospective study was conducted from 1 January 2010 until 31 December 2023 by collaborating with 13 Italian institutions specialized in gynaecologic oncology and was performed according to ethical guidelines. Charts of patients undergoing fertility-sparing treatment for uterine tumours and with a histological diagnosis of STUMP were retrieved from all centres included in the study. The Institutional Review Board (IRB) of each institution approved retrospective studies (IRB#68/12), and all the patients signed written consent for research purposes. Patients who declined to participate in the study were not included. Inclusion criteria were: (i) histologically proven STUMP; (ii) women who received fertility-sparing management based on their age and reproductive desires; and (iii) age ≥18 and ≤45 years. Exclusion criteria were: (i) consent withdrawn; (ii) history of another cancer or cancer treatment in the last 10 years; and (iii) presence of synchronous solid cancer. The primary objective of this study was to assess the reproductive outcomes of patients who underwent conservative surgery for STUMP. The secondary objective was to analyse the clinical outcome of these patients.

All patients were assessed at baseline by pelvic examination and transvaginal ultrasound (supplemented with a transabdominal scan, if necessary) using a standardized examination technique, as previously described (Harmsen *et al.*, 2022). The American Society of Anaesthesiologists (ASA) score and the Eastern Cooperative Oncology Group (ECOG) scale of performance status were used to conduct all preoperative medical evaluations. All surgeries were performed under general endotracheal anaesthesia. All surgical procedures were performed by consultant surgeons who are experts in reproductive surgeries and gynaecologic oncology. The tumour removal technique was by an open surgery, laparoscopy, or a hysteroscopic approach, according to international guidelines, patients’ characteristics, and tumour localization. The technique for tumour removal was consistent across both open and minimally invasive approaches. Following a vertical incision of the uterine serosa and musculature using monopolar energy, the tumour was accessed and enucleated through traction and countertraction, with blood loss managed via bipolar coagulation and/or ligation. Care was taken to preserve the pseudocapsule, and closure of the myometrial edges was performed in multiple layers using 0 or 1–0 absorbable sutures. If the uterine cavity was entered, the endometrium was repaired using 3–0 absorbable suture. In case of laparoscopy, specimen removal was always obtained by morcellation in an endoscopic retrieval bag or after conversion to an open approach. Hysteroscopic tumour resection was conducted with a resectoscope, utilizing 0.9% normal saline as the distension medium and a bipolar loop for resection.

The digital database, including data of all surgical procedures performed on women enrolled in the study, is maintained at research-grade quality and regularly updated by trained residents in accordance with the standards of the American College of Surgeons’ National Quality Improvement Program (ACS NQIP) platform. Individual records were assessed to select baseline patient and disease characteristics (Hutter *et al.*, 2006).

In each institution, an expert pathologist specialized in gynaecologic oncology performed all histological examinations and revised all cases for the purposes of this study. Histopathological classification was performed according to WHO criteria (Oliva *et al.*, 2014). The classification of tumours was based on mitotic count, type of necrosis, cytologic atypia, and assessment of cell type. STUMPs show morphological features that were exceeded the criteria for usual leiomyoma (and leiomyoma variants) yet are insufficient for a diagnosis of a leiomyosarcoma (Richtarova *et al.*, 2023). The morphological criteria used to define STUMPs were (Ip *et al.*, 2020): (i) spindled smooth muscle tumours: focal/multifocal or diffuse cytologic atypia and 2–4 mitoses/mm^2^ (6–9 mitoses/10 high power fields, 0.55 mm field of diameter, 0.24 mm^2^ in area) but lacking coagulative necrosis; unequivocal coagulative necrosis but lacking cytologic atypia or elevated mitoses; elevated mitoses at >6 mitoses/mm^2^ or >15 mitoses/10 high power fields (field of diameter (FD) = 0.55, 0.24 mm^2^ in area) but lacking coagulative necrosis or cytologic atypia; diffuse cytologic atypia and uncertain mitotic count, often due to prominent karyorrhexis but lacking coagulative necrosis; (ii) epithelioid smooth muscle tumours: epithelioid morphology with 2–3 mitoses/10 high power fields (FD = 0.55, 0.24 mm^2^ in area) but lacking moderate to severe cytologic atypia and coagulative necrosis; and (iii) myxoid smooth muscle tumours: myxoid morphology but lacking mitotic activity, moderate to severe cytologic atypia, coagulative necrosis and infiltrative/irregular borders.

Follow-up evaluations were scheduled every 6 months for the first 5 years after surgery and annually thereafter. The follow-up regimen comprised consultation, vaginal and abdominal ultrasound, and clinical examination. A chest CT scan was performed annually to exclude recurrence. Survival data were abstracted from a dedicated database prospectively updated regularly. Rigorous efforts, including telephonic interviews, were made to improve quality of follow-up data. Duration of follow-up was counted from date of surgery to last follow-up or date of death. Disease-free survival was defined as the time from the day of surgery to uterine mass recurrence, while overall survival was defined as the time from the day of surgery to death.

### Statistical analysis

Statistical analyses were performed using IBM-Microsoft SPSS (SPSS Statistics, IBM Corp., Armonk, NY, USA, 2013) version 20.0 for Mac. Data are summarized using basic descriptive statistics. The categorical variables were depicted as numbers or percentages, and the numerical variables were elicited as mean and SD or median and range. Incidence of events among the groups was analysed for statistical significance by using the Pearson’s test.

## Results

A total of 106 patients were included in this study; the distribution according to institution recruitment is summarized in Table 1. The main characteristics of the study population are reported in Table 2. Most patients underwent open surgery (69.8%), while laparoscopy and hysteroscopy were less common (20.8% and 9.4%, respectively). The median (range) length of the study population follow-up was 48 (7–191) months.

**Table 1.** Distribution of patients according to recruitment at institutions in Italy.

Institution	City	Number of patients included in the study
Malzoni Research Hospital	Avellino	5
S. Orsola-Malpighi Hospital	Bologna	6
IRCCS Ospedale Policlinico San Martino	Genoa	2
Fondazione IRCCS Istituto Nazionale dei Tumori	Milan	22
European Institute of Oncology IRCCS	Milan	5
Fondazione IRCCS Ca' Granda Ospedale Maggiore Policlinico	Milan	7
Fondazione IRCCS San Gerardo dei Tintori Hospital	Monza	7
Padova University Hospital	Padova	6
Fondazione Policlinico Universitario A. Gemelli IRCCS	Rome	33
S. Anna Hospital	Turin	8
‘Filippo Del Ponte’ Hospital	Varese	3
IRCCS Sacred Heart Hospital Don Calabria	Negrar (Verona)	1
Azienda Ospedaliera Universitaria Integrata	Verona	1
Total		106

**Table 2.** Main characteristics of the study population.

	**Study population** (n = 106)
**Age at diagnosis** (years; mean ± SD)	35.3 ± 6.8
**Body mass index** (kg/m^2^; mean ± SD)	24.1 ± 4.3
**Parity** (median, range)	0 (0–3)
**Previous hormonal treatment** (n, %)	47 (44.3)
Estro-progestin	34 (72.3)
Progestin	9 (19.1)
Progestin intrauterine device	2 (4.3)
Not available	2 (4.3)
**Hormonal therapy at time of surgery** (n, %)	24 (22.6)
Estro-progestin	18 (75.0)
Progestin	5 (20.8)
Progestin intrauterine device	1 (4.2)
Not available	0 (0)
**Number of uterine lesions** (median, range)	1 (1–6)
**Maximum diameter of STUMP** (cm; median, range)	7.0 (1–40)
**Type of surgery** (n, %)	
Minimally invasive	22 (20.8)
Open	74 (69.8)
Hysteroscopy	10 (9.4)

STUMP, uterine smooth muscle tumours with uncertain malignant potential.

### Reproductive outcomes

Table 3 shows the characteristics of the reproductive history of the patients. There were 24 (22.6%) patients who had at least one pregnancy before STUMP surgery. Of these 24 women, 14 (58.3%) had previous live births. Table 4 summarizes the main reproductive outcomes of the study population. There were 47 patients (44.3%) who actively tried to conceive after conservative surgery, and 27 (57.4%) of them achieved a pregnancy. Caesarean section was the most frequent type of delivery (82.6%), while the remainder of the cases had a vaginal delivery (17.4%). Among patients trying to conceive, 12 (25.5%) women had more than one pregnancy after surgery for STUMP.

**Table 3.** Characteristics of pregnancies preceding STUMP surgery.

	**Study population** (n = 106)
**Patients with a previous pregnancy** (n, %)	
No	82 (77.4)
Yes	24 (22.6)
**Number and characteristics of previous pregnancies**	
Miscarriages	6 (25.0)
Voluntary termination of pregnancy	3 (12.5)
Preterm delivery	1 (4.2)
Term delivery	14 (58.3)
**Previous caesarean section** (n, %)	3 (21.4)
**Modality of conception** (n, %)	
Natural	22 (91.7)
ART	2 (8.3)

STUMP, uterine smooth muscle tumours with uncertain malignant potential.

**Table 4.** Reproductive outcomes of the study population.

	**Study population** (n = 106)
**Patients who actively tried to conceive** (n, %)	47 (44.3)
**Time between surgery and offspring search** (months; median, range)	11.5 (2–110)
**Age at offspring search** (years; mean ± SD)	35.6 ± 5.4
**Patients achieving a pregnancy** (n, %)	27 (57.4)
**Modality of conception** (n, %)	
Natural	21 (77.8)
ART	6 (22.2)
**Characteristics of pregnancies** (n, %)	
Miscarriage	2 (7.4)
Termination of pregnancy	1 (3.7)
Fetal death in utero	1 (3.7)
Preterm delivery	2 (7.4)
Term delivery	21 (77.8)
**Type of delivery** (n, %)	
Spontaneous vaginal delivery	4 (17.4)
Assisted vaginal delivery (forceps or vacuum device)	0 (0.0)
Caesarean section	19 (82.6)
**Pregnancy complications** (n, %)	
None	22
Gestational diabetes	2
Hypothyroidism pregnancy	1
Intrauterine growth restriction	1
Risk of preterm birth	1
**Additional pregnancies after the first achieved after STUMP surgery** (n)	12
**Type of delivery in additional pregnancies** (n, %)	
Spontaneous vaginal delivery	0 (0.0)
Assisted vaginal delivery (forceps or vacuum device)	0 (0.0)
Caesarean section	12 (100.0)

STUMP, uterine smooth muscle tumours with uncertain malignant potential.

### Clinical outcomes

Out of 106 women, 23 (21.7%) had a recurrence of uterine disease. The median (range) time to recurrence in the study population was 18 (4–129) months. The main features of clinical outcomes are shown in Table 5. No difference in the rate of recurrence was reported according to the surgical technique (*P* = 0.210). Furthermore, a higher rate of recurrence was observed among patients who became pregnant (17 out of 27 women (63.0%)) compared with those who did not (6 out of 79 women (7.6%); *P* < 0.001).

**Table 5.** Clinical outcomes of the study population.

	**Study population** (n = 106)
**Recurrences** (n, %)	23 (21.7)
**Time between first STUMP diagnosis and uterine disease recurrence** (months; median, range)	18 (4–129)
**Diagnostic technique** (n)*	
Ultrasound	18
CT	4
MRI	5
PET	1
**Histology of recurrence** (n, %)	
Leiomyomas	10 (43.5)
STUMP	11 (47.9)
Leiomyosarcoma	1 (4.3)
Low-grade leiomyosarcoma	1 (4.3)
**Site of recurrence** (n, %)	
Pelvic	21 (91.3)
Extra-pelvic	2 (8.7)
Distant	0 (0.0)
**Surgery at the recurrence** (n, %)	21 (91.3)
**Type of surgery at recurrence** (n, %)	
Hysteroscopic myomectomy	3 (14.3)
Laparotomy myomectomy	7 (33.3)
Laparotomy hysterectomy	3 (14.3)
Laparoscopy hysterectomy	8 (38.1)
**Demolitive surgery during follow-up for any reason** (n, %)	14 (13.2)
After having achieved pregnancy	4 (3.8)
Without having achieved a pregnancy	10 (9.4)
**Follow-up** (months; mean, range)	48 (7–191)
**Status** (n, %)	
Alive	105 (99.1)
Death of disease	1 (0.9)
Death of other causes	0 (0.0)

* One patient could be studied by more than one imaging technique.

CT, computed tomography; PET, positron emission tomography; STUMP, uterine smooth muscle tumours with uncertain malignant potential.

Recurrence mainly occurred in the pelvis (91.3%), while extrapelvic relapse was reported in a minority of cases (8.7%). Histology of recurrence was represented by leiomyomas and STUMP in 43.5% and 47.9% of the cases, respectively. Only two cases (1.9%) of malignant relapse were recorded, namely one leiomyosarcoma of the uterus and one abdominal low-grade leiomyosarcoma. At follow-up, the patient who had the leiomyosarcoma recurrence died after being treated with radical surgery and chemotherapy. In contrast, the patient who had a low-grade leiomyosarcoma recurrence is still alive. Fourteen (13.2%) patients underwent total hysterectomy after fertility-sparing treatment. Four (3.8%) women had their uterus removed after having completed their reproductive plans, while 10 (9.4%) patients underwent hysterectomy at recurrence without having achieved a pregnancy.

## Discussion

### Summary of main results

Based on the findings of this multicentre retrospective study, patients diagnosed with STUMP can be effectively and safely managed with fertility-sparing treatment. Approximately half of the study population attempted to conceive following conservative surgery for STUMP, and nearly 60% of these patients successfully achieved at least one pregnancy. Additionally, the recurrence rate was 22%, with the majority of relapses involving benign disease or STUMP, and only two cases progressing to malignancy. According to our data analysis, only one patient had died by the time of follow-up.

### Results in the context of published literature

STUMPs are a rare finding in gynaecology, with their precise incidence yet to be fully established (Gadducci and Zannoni, 2019). They are diagnosed when a smooth muscle tumour fails to meet the criteria for leiomyosarcoma but exhibits one or more concerning features (Oliva *et al.*, 2014). The symptoms and signs of STUMP closely resemble those of leiomyomas, including pelvic pain, abnormal uterine bleeding, anaemia, dysmenorrhoea, pelvic mass, infertility, and symptoms caused by compression of adjacent organs, making differential diagnosis a clinical challenge. Additionally, imaging techniques like ultrasound and diffusion-weighted magnetic resonance imaging show limited accuracy in identifying smooth muscle uterine tumours (Gadducci and Zannoni, 2019), although recent advancements in risk stratification strategies have shown some promise (Ciccarone *et al.*, 2025). At diagnosis, the median age of patients with STUMP is 41–48 years, ranging from 20 to 75 years (Gadducci and Zannoni, 2019). More frequently, STUMPs are slow-growing tumours associated with an overall good prognosis even if, rarely, they can relapse and metastasize as malignant. However, most of the patients with STUMP have a long clinical course after recurrence (Gadducci and Zannoni, 2019). Disease-free and overall survival range between 66–80% and 92–100%, respectively (Guntupalli *et al.*, 2009; Ng *et al.*, 2010; Gadducci and Zannoni, 2019; Di Giuseppe *et al.*, 2022). It should be considered that in the last decades, a relevant shift to pregnancy at older maternal ages has been reported, particularly in high-income countries. Given this background, accurate counselling should always be offered to selected patients of fertile age with a diagnosis of a suspicious uterine mass, considering uterus-saving procedures (Ray-Coquard *et al.*, 2024). Data on the fertility-sparing management of women with a diagnosis of STUMP are scanty, and the available studies include small populations of patients (Campbell *et al.*, 2016; Ha *et al.*, 2018; Karataşlı *et al.*, 2019; Şahin *et al.*, 2019; Huo *et al.*, 2020; Shim *et al.*, 2020; Ning *et al.*, 2021; Zhang *et al.*, 2021; Richtarova *et al.*, 2023; Garg *et al.*, 2024). In 2015, Campbell *et al.* reported the first report of pregnancy after the diagnosis of a STUMP treated with myomectomy (Campbell *et al.*, 2016). Since this report, reproductive outcomes of patients with STUMP who received fertility-sparing treatment have been poorly investigated. Almost all previous studies included women, not only in their reproductive years, who were treated by either radical or conservative surgery, and these investigations were not specifically designed to assess the reproductive desires and outcomes of the patients (Ha *et al.*, 2018; Karataşlı *et al.*, 2019; Şahin *et al.*, 2019; Huo *et al.*, 2020; Shim *et al.*, 2020; Ning *et al.*, 2021; Zhang *et al.*, 2021; Garg *et al.*, 2024). Şahin *et al.* conducted a dual-institutional study to evaluate the clinicopathological features and obstetric and oncological outcomes of 57 patients diagnosed with STUMPs. In this study, 10 (17.5%) had fertility desire and 7 (70.0%) pregnancies were recorded. One of the conceptions occurred with ART, and another four were natural. Six full-term infants and another live birth at 35 weeks were recorded (Şahin *et al.*, 2019). Recently, Richtarova *et al.* conducted a study with a similar design. The aim was to evaluate the reproductive and clinical outcomes of 46 patients treated with myomectomy who were histologically diagnosed with STUMP. Of 42 (91.3%) patients with unfinished reproductive plans, 22 pregnancies were recorded among 17 women (40.5%), which resulted in 18 uncomplicated deliveries. Unfortunately, no information on the exact number of women who actively tried to achieve a pregnancy was available. Similarly to our study, the rate of caesarean section was very high (17/22 pregnancies, 77.3%), and the rate of hysterectomy after having achieved a pregnancy was relatively low (2/17 women, 11.7%) (Richtarova *et al.*, 2023).

The recurrence rate in our study (21.7%) aligns with that in existing literature. In the previously cited study by Richtarova *et al.* including 46 patients treated for STUMP, 13 (28.3%) reinterventions (five myomectomies and eight hysterectomies) were recorded with benign histology in 11 cases and STUMP histology in two cases (4.3% of all patients). No malignancy or deaths were reported in the study (Richtarova *et al.*, 2023). A 2022 systematic review including 189 patients surgically treated for STUMP demonstrated a recurrence rate of 21.5% (Di Giuseppe *et al.*, 2022). Another systematic review showed that recurrence ranges between 0% and 36.4%, with a mean value of 12.9% and a median time to recurrence of ∼51 months (range, 15 months–9 years). However, it should be considered that this systematic review did not include only studies investigating fertility-sparing treatment but also treatment by direct radical surgery. Therefore, it is likely that the relevant recurrence rate could be lower when compared to the results of our study (Gadducci and Zannoni, 2019). Nevertheless, accurately determining the true recurrence rate is challenging due to various biases in the available evidence, including inconsistent diagnostic criteria, small patient cohorts, and varying follow-up durations. In our study, most patients who had surgery following the fertility-sparing treatment had histologically proven benign disease (leiomyoma) in 43.5% of the cases, STUMP in 47.9%, and a malignancy in 8.6%. Furthermore, in most of the cases, recurrence occurred in the pelvis (91.3%) while extrapelvic disease was reported in less than 10%. Several studies are consistent with these data, showing that recurrence after conservative surgery is more frequently represented by pelvic diseases such as leiomyomas or STUMPs (Campbell *et al.*, 2016; Ha *et al.*, 2018; Gadducci and Zannoni, 2019; Karataşlı *et al.*, 2019; Şahin *et al.*, 2019; Huo *et al.*, 2020; Shim *et al.*, 2020; Ning *et al.*, 2021; Zhang *et al.*, 2021; Richtarova *et al.*, 2023; Garg *et al.*, 2024). Available data demonstrated that the recurrence rate of malignant disease ranges from 0 to ∼30% (Di Giuseppe *et al.*, 2022). Interestingly, our study showed that patients who became pregnant had a higher recurrence rate than those who did not. However, this information was not evaluated in previous studies on the fertility-sparing treatment of STUMP (Gadducci and Zannoni, 2019; Şahin *et al.*, 2019; Richtarova *et al.*, 2023).

### Strengths and limitations

This study’s retrospective design introduces inherent biases, yet it benefits from the participation of tertiary referral centres for gynaecologic oncology. Counselling, presurgical diagnostics, and surgical management were performed by specialists in gynaecologic malignancies, all histological analyses were conducted by experienced pathologists, and all cases were reviewed specifically for this study. Efforts were made to ensure complete and accurate data collection, including follow-up updates.

Importantly, this is the largest case series to date examining the reproductive and clinical outcomes of fertility-sparing treatment in STUMP patients, focusing exclusively on women desiring future pregnancies. These findings provide valuable guidance for clinicians counselling patients about fertility-sparing treatment, which should be considered both effective and safe.

## Conclusion

This multicentre retrospective study confirms that fertility-sparing management for patients diagnosed with STUMP is both feasible and safe. However, we strongly recommend that such treatment be conducted in specialized centres with expertise in gynaecologic oncology. These centres should provide a multidisciplinary approach encompassing high-quality diagnostic evaluation, surgical management, pathological analysis, and comprehensive fertility counselling.

## Data Availability

Data are available on request.

## References

[hoaf009-B1] Baird DD , Dunson DB, Hill MC, Cousins D, Schectman JM. High cumulative incidence of uterine leiomyoma in black and white women: ultrasound evidence. Am J Obstet Gynecol 2003;188:100–107. 12548202 10.1067/mob.2003.99

[hoaf009-B2] Bell SW , Kempson RL, Hendrickson MR. Problematic uterine smooth muscle neoplasms. A clinicopathologic study of 213 cases. Am J Surg Pathol 1994;18:535–558. 8179071

[hoaf009-B3] Borella F , Mancarella M, Preti M, Mariani L, Stura I, Sciarrone A, Bertschy G, Leuzzi B, Piovano E, Valabrega G et al Uterine smooth muscle tumors: a multicenter, retrospective, comparative study of clinical and ultrasound features. Int J Gynecol Cancer 2024;34:244–250. 38054268 10.1136/ijgc-2023-004880

[hoaf009-B4] Bucuri CE , Ciortea R, Malutan AM, Oprea V, Toma M, Roman MP, Ormindean CM, Nati I, Suciu V, Mihu D. Smooth muscle tumor of uncertain malignant potential (STUMP): a systematic review of the literature in the last 20 years. Curr Oncol 2024;31:5242–5254. 39330016 10.3390/curroncol31090388 PMC11430651

[hoaf009-B5] Campbell JE , Knudtson JF, Valente PT, Robinson RD, Kost ER. Successful pregnancy following myomectomy for uterine smooth muscle tumor of uncertain malignant potential: a case report and review of the literature. Gynecol Oncol Rep 2016;15:1–3. 26937476 10.1016/j.gore.2015.07.005 PMC4750020

[hoaf009-B6] Chiappa V , Interlenghi M, Salvatore C, Bertolina F, Bogani G, Ditto A, Martinelli F, Castiglioni I, Raspagliesi F. Using rADioMIcs and machine learning with ultrasonography for the differential diagnosis of myometRiAL tumors (the ADMIRAL pilot study). Radiomics and differential diagnosis of myometrial tumors. Gynecol Oncol 2021;161:838–844. 33867144 10.1016/j.ygyno.2021.04.004

[hoaf009-B7] Ciccarone F , Biscione A, Robba E, Pasciuto T, Giannarelli D, Gui B, Manfredi R, Ferrandina G, Romualdi D, Moro F et al A clinical ultrasound algorithm to identify uterine sarcoma and smooth muscle tumors of uncertain malignant potential in patients with myometrial lesions: the MYometrial Lesion UltrasouNd And mRi study. Am J Obstet Gynecol 2025;232:108.e1–108.e22. 10.1016/j.ajog.2024.07.027 39084498

[hoaf009-B8] Devereaux KA , Schoolmeester JK. Smooth muscle tumors of the female genital tract. Surg Pathol Clin 2019;12:397–455. 31097110 10.1016/j.path.2019.02.004

[hoaf009-B9] Di Giuseppe J , Grelloni C, Giuliani L, Delli Carpini G, Giannella L, Ciavattini A. Recurrence of uterine smooth muscle tumor of uncertain malignant potential: a systematic review of the literature. Cancers 2022;14:2323. 35565452 10.3390/cancers14092323 PMC9104240

[hoaf009-B11] Gadducci A , Zannoni GF. Uterine smooth muscle tumors of unknown malignant potential: a challenging question. Gynecol Oncol 2019;154:631–637. 31326137 10.1016/j.ygyno.2019.07.002

[hoaf009-B12] Garg M , Rajanbabu A, Nair IR. Smooth muscle tumors of uncertain malignant potential or atypical leiomyomas: a long-term evaluation of surgical outcomes and clinicopathological features. Eur J Obstet Gynecol Reprod Biol 2024;301:201–205. 39154515 10.1016/j.ejogrb.2024.07.062

[hoaf009-B13] Guntupalli SR , Ramirez PT, Anderson ML, Milam MR, Bodurka DC, Malpica A. Uterine smooth muscle tumor of uncertain malignant potential: a retrospective analysis. Gynecol Oncol 2009;113:324–326. 19342083 10.1016/j.ygyno.2009.02.020 PMC8248445

[hoaf009-B14] Ha HI , Choi MC, Heo JH, Kim KA, Jung SG, Park H, Joo WD, Song SH, Kim TH, Lee C. A clinicopathologic review and obstetric outcome of uterine smooth muscle tumor of uncertain malignant potential (STUMP) in a single institution. Eur J Obstet Gynecol Reprod Biol 2018;228:1–5. 29902779 10.1016/j.ejogrb.2018.06.003

[hoaf009-B15] Harmsen MJ , Van den Bosch T, de Leeuw RA, Dueholm M, Exacoustos C, Valentin L, Hehenkamp WJK, Groenman F, De Bruyn C, Rasmussen C et al Consensus on revised definitions of Morphological Uterus Sonographic Assessment (MUSA) features of adenomyosis: results of modified Delphi procedure. Ultrasound Obstet Gynecol 2022;60:118–131. 34587658 10.1002/uog.24786 PMC9328356

[hoaf009-B16] Huo L , Wang D, Wang W, Cao D, Yang J, Wu M, Yang J, Xiang Y. Oncologic and reproductive outcomes of uterine smooth muscle tumor of uncertain malignant potential: a single center retrospective study of 67 cases. Front Oncol 2020;10:647. 32477938 10.3389/fonc.2020.00647 PMC7240040

[hoaf009-B17] Hutter MM , Rowell KS, Devaney LA, Sokal SM, Warshaw AL, Abbott WM, Hodin RA. Identification of surgical complications and deaths: an assessment of the traditional surgical morbidity and mortality conference compared with the American College of Surgeons-National Surgical Quality Improvement Program. J Am Coll Surg 2006;203:618–624. 17084322 10.1016/j.jamcollsurg.2006.07.010

[hoaf009-B18] Ip P , Croce S, Gupta M. Smooth muscle tumour of uncertain malignant potential of the uterine corpus. In: WHO Classification of Tumours Editorial Board (ed). WHO Classification of Tumours of Female Genital Tumours. Lyon: Internal Agency for Research on Cancer (IARC), 2020, 279–280.

[hoaf009-B19] Karataşlı V , Çakır İ, Ayaz D, Budak A, Sancı M. Clinicopathologic evaluation of uterine smooth muscle tumors of uncertain malignant potential (STUMP): a single center experience. J Gynecol Obstet Hum Reprod 2019;48:637–642. 30898630 10.1016/j.jogoh.2019.03.003

[hoaf009-B20] Mathews TJ , Hamilton BE. *First births to older women continue to rise*. NCHS data brief, no. 152. Hyattsville, MD, USA: National Center for Health Statistics, 2014. 24813228

[hoaf009-B21] Ng JS , Han A, Chew SH, Low J. A clinicopathologic study of uterine smooth muscle tumours of uncertain malignant potential (STUMP). Ann Acad Med Singap 2010;39:625–628. 20838704

[hoaf009-B22] Ning C , Zhang L, Zhao C, Chen X, Liu X, Gu C. Clinical and reproductive outcomes of uterine smooth muscle tumor of uncertain malignant potential: a single-center retrospective study. J Int Med Res 2021;49:3000605211008065. 33884911 10.1177/03000605211008065 PMC8074534

[hoaf009-B23] Oliva E , Carcangiu ML, Carinelli SG, Ip P, Loening T, Longacre TA, Nucci MR, Prat J, Zaloudek C. Mesenchymal tumours. Smooth muscle tumour of uncertain malignant potential. In: Kurman RJ, Carcangiu ML, Herrington CS, Young RH (eds). WHO Classification of Tumours of Female Reproductive Organs. Lyon: Internal Agency for Research on Cancer (IARC), 2014, 135–147.

[hoaf009-B24] Ray-Coquard I , Casali PG, Croce S, Fennessy FM, Fischerova D, Jones R, Sanfilippo R, Zapardiel I, Amant F, Blay JY et al ESGO/EURACAN/GCIG guidelines for the management of patients with uterine sarcomas. Int J Gynecol Cancer 2024;34:1499–1521. 39322612 10.1136/ijgc-2024-005823

[hoaf009-B25] Richtarova A , Boudova B, Dundr P, Lisa Z, Hlinecka K, Zizka Z, Fruhauf F, Kuzel D, Slama J, Mara M. Uterine smooth muscle tumors with uncertain malignant potential: analysis following fertility-saving procedures. Int J Gynecol Cancer 2023;33:701–706. 36898699 10.1136/ijgc-2022-004038 PMC10176401

[hoaf009-B26] Şahin H , Karatas F, Coban G, Özen Ö, Erdem Ö, Onan MA, Ayhan A. Uterine smooth muscle tumor of uncertain malignant potential: fertility and clinical outcomes. J Gynecol Oncol 2019;30:e54. 31074239 10.3802/jgo.2019.30.e54 PMC6543118

[hoaf009-B27] Shim JI , Han AKW, Jeon HJ, Kim ML, Jung YW, Yun BS, Seong SJ, Shin E, Cho YJ, Rha SH. Clinical experience of uterine smooth muscle tumor of uncertain malignant potential in two gynecological centers: oncological and obstetrical aspects. Eur J Obstet Gynecol Reprod Biol 2020;246:7–13. 31927240 10.1016/j.ejogrb.2020.01.002

[hoaf009-B28] Zhang C , Gao J, Lu S, Zhang Y, Zhu H. Uterine smooth muscle tumors of uncertain malignant potential (STUMP): A retrospective study in a single center. Eur J Obstet Gynecol Reprod Biol 2021;265:74–79. 34467879 10.1016/j.ejogrb.2021.08.010

[hoaf009-B29] Zimmermann A , Bernuit D, Gerlinger C, Schaefers M, Geppert K. Prevalence, symptoms and management of uterine fibroids: an international internet-based survey of 21,746 women. BMC Womens Health 2012;12:6. 22448610 10.1186/1472-6874-12-6 PMC3342149

