# Correction to: Uterine smooth muscle tumours with uncertain malignant potential: reproductive and clinical outcomes in patients undergoing fertility-sparing management

**DOI:** 10.1093/hropen/hoaf032

**Published:** 2025-06-03

This is a correction to: Umberto Leone Roberti Maggiore, Francesco Fanfani, Giovanni Scambia, Ilaria Capasso, Emanuele Perrone, Giuseppe Parisi, Gian Franco Zannoni, Francesca Falcone, Alessandra Di Giovanni, Mario Malzoni, Anna Myriam Perrone, Francesco Mezzapesa, Pierandrea De Iaco, Simone Garzon, Pier Carlo Zorzato, Stefano Uccella, Fabio Barra, Stefano Bogliolo, Simone Ferrero, Veronica Iannuzzi, Dorella Franchi, Tommaso Bianchi, Tommaso Grassi, Robert Fruscio, Giulia Vittori Antisari, Giovanni Roviglione, Marcello Ceccaroni, Fulvio Borella, Stefano Cosma, Alberto Revelli, Jvan Casarin, Anna Giudici, Fabio Ghezzi, Matteo Marchetti, Giulia Spagnol, Roberto Tozzi, Francesca Filippi, Michela Molgora, Giovanna Scarfone, Biagio Paolini, Stefano Fucina, Valentina Chiappa, Antonino Ditto, Giorgio Bogani, Francesco Raspagliesi, Uterine smooth muscle tumours with uncertain malignant potential: reproductive and clinical outcomes in patients undergoing fertility-sparing management, *Human Reproduction Open*, Volume 2025, Issue 2, 2025, hoaf009, https://doi.org/10.1093/hropen/hoaf009

In the originally published version of the article, the following acknowledgement was omitted from the funding statements in the extended abstract and at the end of the article: ‘This work was supported by Ricerca Corrente funds, Italian Ministry of Health.’

The published article has been updated to include this funding source.

